# The Effects of Lime and Cement Addition on the Compaction and Shear Strength Parameters of Silty Soils

**DOI:** 10.3390/ma18050974

**Published:** 2025-02-21

**Authors:** Andrzej Gruchot, Katarzyna Kamińska, Agnieszka Woś

**Affiliations:** Department of Hydraulic Engineering and Geotechnics, University of Agriculture in Kraków, Mickiewicza 24/28, 30-059 Kraków, Poland; katarzyna.kaminska@urk.edu.pl (K.K.); agnieszka.wos@urk.edu.pl (A.W.)

**Keywords:** cement, lime, silty soil, stabilization, compaction parameters, shear strength, extreme gradient boosting (XGBoost), SHAP value

## Abstract

This article presents the results of laboratory tests of compaction parameters and shear strength of silty soils with and without the addition of hydraulic binder in the form of lime and/or cement. The tests were carried out on samples formed with an optimum moisture content and with 0, 3, 5, and 8% hydraulic binder added to the dry mass of the soil. The soil samples were examined after 7 and 14 days of air–water treatment without and with freeze–thaw cycles. It was found that the addition of lime and cement caused changes in the compaction parameters. This effect depended to a large extent on the type of binder, and also on the grain size composition of the tested soil. The tests showed that the shear strength and the parameters describing it, i.e., the angle of internal friction and cohesion, were high and largely depended on the type of binder and the sample treatment method, as well as its duration. The obtained results indicate that the use of hydraulic binders was an effective method of surface stabilization. Improving soil properties based on the addition of a hydraulic binder is a beneficial method for the environment from the viewpoint of sustainable development and reducing CO_2_ emissions because it does not require the use of, e.g., soil replacement. Using the SHAP algorithm, it was found that normal stress, initial moisture content, and curing time of the samples were the main input features that influenced the shear strength.

## 1. Introduction

In civil engineering earthworks, the deficit of land suitable for the implementation of specific goals related to road or hydrotechnical construction is increasingly noticed. In the case of road construction, there is a need to move significant earth masses, and in many cases, this results in negative balance of earth masses for a given road section. This necessitates bringing in soil from outside the road area. In such situations, guided by economic criteria, deposits of various materials are used. In many cases, these are anthropogenic soils, i.e., materials created as a result of human industrial activity—metallurgical slag, ash–slag mixtures, fly ash and coal shales, foundry sands, as well as reclaimed concrete [1,2,3,4]. Another method is deep or surface soil stabilization using hydraulic binders [3,5,6] and/or the addition of dispersed reinforcement [7,8,9,10] or stabilization through soil granulation, i.e., changing the grain size composition.

The concept of soil reinforcement by mixing it with a hydraulic binder is widely used in civil engineering earthworks, especially in road construction. For this purpose, various methods of surface soil stabilization are most often used, mainly the addition of lime, cement, fly ash, and blast furnace slag. Generally, the effects of reinforcement are observed in all soils mixed with a hydraulic binder, but the effectiveness of reinforcement is highly diversified and decreases especially in organic soils. A characteristic feature of soils stabilized for road purposes is the relatively low conversion costs of the binder and the fact that mixing is conducted “in dry conditions”. Mixing the soil with the binder is currently performed using specialist machines that dose the appropriate amount of binder and mix it with the soil. For many years, research has focused on surface stabilization of soils using lime, cement, fly ash, fine fractions of industrial waste, phosphogypsum, potassium nitrate, calcium chloride, and phosphoric acid [11,12,13,14,15,16,17,18,19,20,21,22,23,24]. The addition of a few percent by weight of cement or lime has been shown to be effective in improving workability and compaction, as well as providing significant savings compared to the cost of removing and replacing low-bearing soil. However, the effectiveness and improvement of engineering properties requires systematic testing for design and construction.

The use of hydraulic binders such as lime, cement, fly ash, and other dedicated hydraulic binders, most often based on lime or cement, allows for the improvement of geotechnical properties of mineral soils occurring in the subsoil or used as a material for the construction of earth embankments. Soils that require stabilization are most often characterized by low strength and poor workability, which is a problem in the implementation of construction projects. Soil improvement techniques are increasingly used and modified depending on the required end result (e.g., improvement of compaction, limitation of consolidation, increase in bearing capacity), which can be predicted using appropriate numerical modeling techniques (e.g., [25]). In most cases, soils are treated to increase shear strength, bearing capacity, stability, and deformation control. The choice of a binder and the effectiveness of the amount of its addition depend on the type of soil and field conditions; however, knowledge of the mechanical behavior of the treated soil has a significant impact on the choice of stabilizer. The obtained effect may comprise the absorption and chemical binding of water, which facilitates compaction and reduces plasticity of soils [26]. On the other hand, the intensive development of soil strengthening methods is associated with the search for cheaper but effective methods of strengthening, taking into account the requirements of environmental protection and sustainable economy. Another important factor is the desire to shorten the construction cycle of new facilities, which means that cheaper but time-consuming geotechnical solutions are not selected for implementation.

Lime is one of the oldest and most popular additives used for soil stabilization [11]. The addition of lime to cohesive soils reduces the liquid limit and increases the plastic limit, which results in a lower plasticity index [27] and allows for higher soil workability. On the other hand, the addition of lime reduces soil moisture. It also increases the optimum moisture content and reduces the maximum dry density of the soil structure, causing a rapid increase in the soil strength [11,27]. It should be clearly stated that the effects of lime depend to a large extent on the type of soil, its grain size and mineralogical structure, and the shape of its particles [28,29]. The proposed addition of lime to the dry mass of soil ranges from 2 to 8% [30]. For example, Pereira et al. [31] used 2% addition of lime to stabilize the subgrade of a forest road in Brazil. The results of the study showed that the addition of lime significantly changed the mechanical properties of the lateritic soil by increasing its mechanical strength and load-bearing capacity. The highest strength values were obtained for the soil–lime mixture after 28 days of treatment. CBR load-bearing index studies showed that the soil–lime mixture after 28 days could be used as a subgrade material for flexible road surfaces, being an alternative to use in the layers of forest roads.

The use of cement as a stabilizer improves soil strength through the same type of pozzolanic reaction that occurs during lime stabilization. Both lime and cement contain calcium, which is necessary for pozzolanic reactions, but the source of silica necessary for pozzolanic reactions is different. In lime stabilization, silica is supplied when clay soil particles disintegrate. In contrast, cement already contains silica without the need to decompose the clay mineral. Thus, unlike lime stabilization, cement stabilization is rather independent of soil properties. Cement-based soil stabilization develops from cementitious bonds between calcium silicate, aluminate hydration products, and soil particles [11,32,33]. The basic requirement is adequate water content to initiate the hydration process. Similarly to lime stabilization, carbonation can also occur when using cement stabilization.

The beneficial effects of cement application on improving the geotechnical properties of a broad range of soils have been widely reported in the literature [24,32,34,35,36,37,38,39]. Studies have shown that the addition of cement to cohesive soils reduces the liquid limit, plasticity index, and swelling, and it increases the shrinkage limit and shear strength [40]. It should be noted that the reduction in the plasticity index of cohesive soils is caused by the increase in the plasticity limit, which is largely influenced by the cement content and setting (curing) time [41]. It was also found that the addition of cement increases the optimum moisture content and decreases the maximum bulk density of the soil skeleton [42]. Moreover, the addition of cement causes an immediate decrease in water content [43]. However, the main factor that determines the use of any of the soil stabilization methods is the grain size and mineral composition of the soil, the type of engineering structure, and the soil–water environment in which the soil will work.

The aim of this study was to determine the effects of adding a hydraulic binder in the form of lime and/or cement on the parameters of compaction and shear strength of silty soils. This study was conducted on samples formed with an optimum moisture content with different percentage of hydraulic binder addition in relation to the dry mass of the soil. The stabilized soil samples were tested after 7 and 14 days of air–water treatment and after the same treatment time but with 3 and 14 freeze–thaw cycles. This study was also conducted on samples of silty soils without the addition of hydraulic binder.

## 2. Materials and Methods

This study was conducted using silty soils from the Lesser Poland region (Poland) occurring in the subsurface layer of roads, the properties of which could potentially be improved through stabilization with binders. Silty soils from excavations from a level of about 0.5–0.7 m below the level of natural ground were used for the tests. It was assumed that the bottom of the road surface structure is most often designed at a similar depth, which should correspond to the top layer of the ground subject to stabilization. The tests were carried out using silt collected from a landslide area in Sandomierz, coarse silt collected from the access road to a hotel in Wieliczka, and clay–sand silt collected from the vicinity of the Mateczny Roundabout in Kraków.

The grain size composition was determined via a combined method, using sieve analysis for grains larger than 0.063 mm and hydrometric analysis for particles smaller than 0.063 mm [44]. The specific density was determined using the volumetric flask method in distilled water [45]. The compaction parameters were determined using a Proctor apparatus with a cylinder with a volume of 1.0 dm^3^ at a compaction energy of 0.59 J∙cm^−3^ [46,47].

The shear strength parameters, i.e., the angle of internal friction and cohesion, were determined using a direct shear apparatus on samples with dimensions of 6.0 × 6.0 × 1.9 cm, which were consolidated and sheared at normal stresses of 50, 100, 150, 200, and 250 kPa [48]. The samples were formed directly in the apparatus box to obtain the compaction index CI = 0.92 and 0.98 for silt (Si) from Sandomierz at moisture contents lower (12.0%) and equal to the optimum (14.9%). On the other hand, in the case of coarse silt (CSi) from Wieliczka and clayey silt with sand (saclSi) from Kraków, the samples were formed with and without the addition of a hydraulic binder at the optimal moisture content determined for each binder and its additive in specially prepared molds. The compaction index of coarse silt samples from Wieliczka was CI = 0.95, and for clayey silt with sand from Kraków, CI = 1.00. The consolidation time of the silt samples from Sandomierz formed with a moisture content lower than and equal to the optimum was 10 min, while for the samples additionally subjected to water accumulation for 24 h, it was 2 min. The reduction in the consolidation time was caused by the need to maintain a high soil moisture content during shearing. In the case of the silty soil collected in Wieliczka, the consolidation time was 5 min, and for clayey silt with sand from Kraków, it was 15 min. The shearing speed was 0.1 mm∙min^−1^ for soil from Sandomierz and 0.2 mm∙min^−1^ for soils from Wieliczka and Kraków. The different values of the compaction index and shearing speed adopted in the tests of individual silty soils resulted from the design requirements.

**Figure 1:**
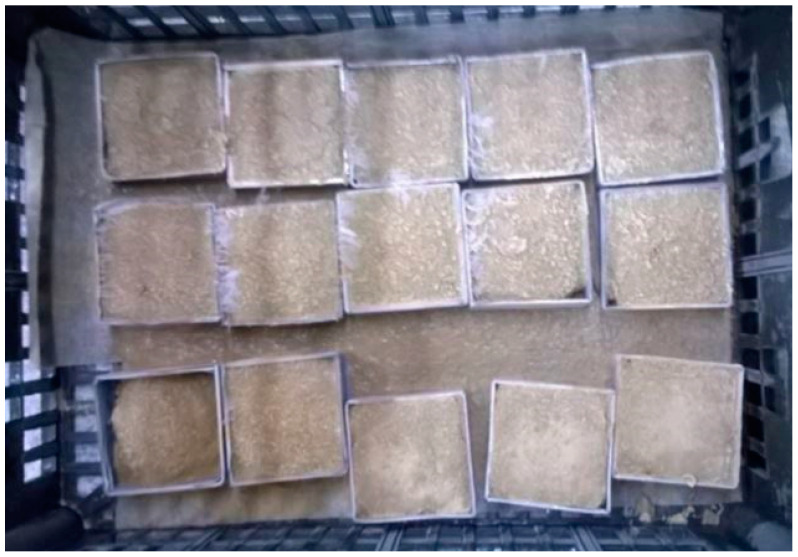
Samples of coarse silt with added cement during treatment after 10 out of 28 days of air–water treatment (photo by K. Kamińska).

The coarse silt was stabilized with hydrated lime [49] and Portland cement CEM I 32.5R [50], while the clayey silt with sand was stabilized with CEM I 32.5R cement in amounts of 3, 5, and 8% of the dry mass of the soil. The samples with the binder addition were sheared immediately after compaction and after 7 and 28 days of air–water treatment (Table 1) [49]. The 7 and 28-day treatments of the samples consisted of storing the formed samples in tightly closed plastic bags for 3 and 13 days, then placing them in a container for one day and filling it with water to a height of 1 cm. For the next 3 and 14 days, the samples were completely immersed in water. On the other hand, the samples subjected to freezing and thawing cycles with 7 and 28-day treatments were stored in tightly closed plastic bags for the initial 3 and 13 days. Then, they were immersed in water for one day, then subjected to 3 and 14 freeze–thaw cycles (Figure 1). A single cycle was 8 h of freezing at −21 °C and 16 h of thawing in water at room temperature.

The obtained test results were subjected to statistical analysis. Based on the correlation matrix, the correlation was determined for independent variables, which were the type of soil and its origin, the content of individual fractions, compaction parameters (maximum bulk density of the skeleton, optimum moisture content), initial and final moisture content, bulk density, dry density, compaction index, and type of binder, as well as type of treatment (air–water, freeze–thaw cycles) and normal stress values. The correlation between shear strength and individual independent parameters was also determined. Each element in the correlation coefficient matrix represents the value of the correlation coefficient between two variables. The correlation coefficients range from −1 to 1, where a value of −1 indicates a strong negative correlation, 1 indicates a strong positive correlation, and 0 indicates no correlation. A strong positive correlation means that when one variable increases, the other variable also increases, while a strong negative correlation means that as one variable increases, the other variable decreases.

In order to determine the influence of individual factors on the shear strength test results, the XGBoost gradient boosting algorithm [51] was used, then the SHAP value for each independent variable was determined. Extreme gradient boosting (XGBoost) is a multi-threaded implementation of decision trees and is a very efficient machine learning algorithm that evolved from traditional machine learning classification and regression trees [52,53]. It is characterized by high efficiency and flexibility in predicting numerical values. It uses numerical data for prediction, but unlike many other popular algorithms, it can also consider qualitative data. XGBoost inputs the original training set into the first regression tree to generate a weak learning model. Then, XGBoost collects the training errors generated by the first weak learning model to build a new dataset with errors. Then, the new dataset is considered as new training data, which are used to train the second regression tree. The above steps are continuously repeated, and the loop ends when the objective function value is less than the desired threshold. The prediction score is the average score of all the trees.

The independent variables were the same factors as in the case of the correlation matrix. In the calculations for model verification, the data were divided into training and test sets in the proportions 75% to 25%. After the model training was completed, the SHAP (Shapley additive explanations) [54] parameter values were calculated for the trained model, which allowed for determining the significance of individual independent parameters on the model prediction results. The sklearn libraries [55] were used to prepare the data.

## 3. Characteristics and Application of Binders

The addition of a hydraulic binder to the soil leads to several key changes that affect the shear strength. Hydraulic binders, when mixed with water, produce hydration products (e.g., calcium sulfate, calcium hydroxide) that create new mineral compounds in the soil structure. This improves its shear strength. The addition of a binder improves the compaction of the soil, which leads to a reduction in the porosity of the material. Increasing the density changes the way the soil particles react to external forces, increasing the resistance to shear. Hydraulic binders create a hard, cohesive mass that improves the soil structure and reduces the susceptibility to deformation under load. This can lead to an in-crease in the bearing capacity of the subsoil and a reduction in the risk of subsidence or settlement.

### 3.1. Lime

Hydrated slaked lime (Ca(OH)_2_) was used in the conducted tests. The applied standard [56] does not provide requirements for the strength of mortar made of hydrated lime. This material is characterized by very low compressive strength, which after 90 days of setting and hardening of the standard mortar does not exceed 1 MPa [57]. Lime production involves heating limestone (CaCO_3_) in order to transform it into high-purity quicklime (CaO), which is associated with the release of carbon dioxide (CO_2_) during the chemical reaction—the so-called “process CO_2_”. This means that lime production is by nature a high-emission process. However, lime captures CO_2_ from its surroundings, thus naturally transforming into limestone. This process is called carbonation, and it reduces the carbon footprint created during its production.

In soil stabilization technology, the most often used material is ground quicklime (CaO), and it can be applied in stabilization or improvement of acidic soils with moisture contents much higher than optimal. The use of hydrated lime (Ca(OH)_2_) is largely devoid of the soil drying effect. However, cohesive soils improved with hydrated lime undergo the flocculation process and, as a result, further binding processes occur in the same way as in the case of quicklime.

Lime reinforcement is most effective in the case of cohesive soils with a plasticity index greater than 10%, a clay fraction content (<0.002 mm) of more than 7%, and a grain size of above 40 mm with less than 15% content. Soils with an organic content above 10% and a sand index above 30 are unsuitable for lime stabilization [58,59]. Lime reinforcement is also not used in even-grained soils due to difficult mixing. Therefore, lime stabilization is mainly used in cohesive fine-grained soils containing a significant amount of clay minerals, but it can also be used in clay gravels and silty and clay sands.

### 3.2. Cement CEM I 32.5R

Portland cement CEM I 32.5 R, with strength class 32.5 [60], was also used in the conducted tests. The standard compressive strength of cement after 28 days ranges from 32.5 to 52.5 MPa, with initial strength equal to or greater than 10 MPa after two days. The composition of Portland cement CEM I is dominated by Portland clinker in the amount of 95 to 100%, with the content of secondary components ranging from 0 to 5%.

Cement stabilization results are the best when it is used as an additive to soils with a sand index of 20 to 50 and a fraction smaller than 0.075 mm content less than 15%, and grains with a diameter greater than 2 mm above 30%. Soils with such parameters are characterized by appropriate porosity, which improves the stabilization process [58].

The mechanism of soil stabilization with cement binder includes binder hydration, ion exchange, formation of cement hydration products, and formation of pozzolanic reaction products. Binder hydration and water absorption reduce the initial moisture content of the stabilized soil, and ion exchange changes the physical properties of the soil, causing a decrease in its plasticity. This phenomenon is used in surface soil stabilization, e.g., in road construction, because adding a small amount of binder facilitates and increases the effectiveness of soil compaction. The phenomena of cement hydration and pozzolanic reaction cause hardening of the soil–cement mixture. The material strength during hydration increases quickly and lasts for a short time, while the pozzolanic reaction is slow and lasts for a long time. As a result, the structure of the hardened soil–cement mixture becomes more compact, forming a material with increased strength and durability [61].

## 4. Study Results

### 4.1. Grain Size Composition and Specific Density

**Figure 2:**
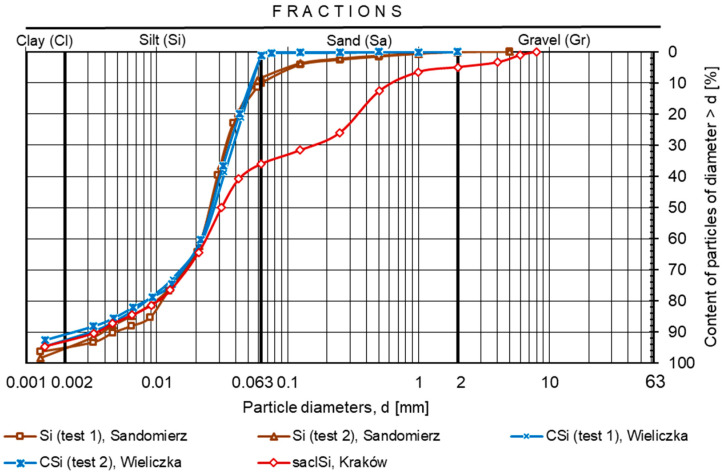
Grain size curve of the tested silty soils.

Based on its grain size composition, the soil from Sandomierz was classified as multi-fractional silt (Si) (Figure 2). The soil was dominated by the silt fraction, which accounted for 86%, and the sand and clay fractions accounted for slightly over 9% and 5%, respectively (Table 2). The soil from Wieliczka was classified as multi-fraction coarse silt (CSi), dominated by the silt fraction in the amount of nearly 88%, whereas the sand and clay fraction contents were slightly more than 4 and 8%, respectively. Finally, the soil from Kraków was classified as sandy clay silt (saclSi). The silt fraction accounted for 57%, the sand fraction accounted for 31%, gravel was 5%, and clay was 7%.

The specific density ranged from 2.67 g∙cm^−3^ for silt and coarse silt to 2.69 g∙cm^−3^ for sand–clay silt (Table 2).

### 4.2. Compaction Parameters

**Figure 3:**
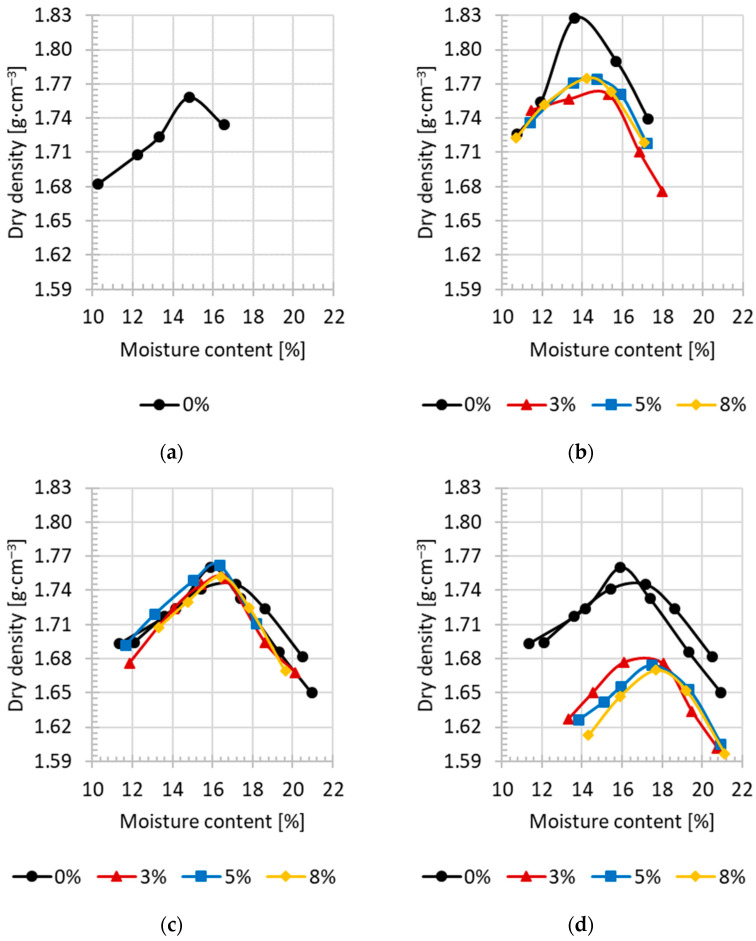
Compaction curves of the tested silty soils with or without hydraulic binder: (**a**) Si, Sandomierz; (**b**) saclSi, Kraków, addition of cement; (**c**) CSi, Wieliczka, addition of lime; (**d**) CSi, Wieliczka, addition of cement.

The compaction parameters of the tested silty soils with and without the addition of lime and/or cement were determined using a standard Proctor apparatus (Figure 3 and Table 3). The dependence of the soil dry density on the moisture content for the soils with the addition of hydraulic binder was similar to that for the soil without the addition of binder. The values of the compaction parameters depended on the amount of lime or cement added to the soil. Generally, the maximum dry density of the soil skeleton decreased and the optimum moisture content increased with the increase in the amount of added binder.

The maximum dry density of the silt without the addition of binder was 1.76 g∙cm^−3^ at an optimum moisture of 14.9% (Figure 3a). In the case of coarse silt, the maximum dry density was on average 1.75 g∙cm^−3^, and the optimum moisture content was 16.4% (Figure 3c). On the other hand, the maximum dry density of coarse silt with the addition of lime was on average 1.68 g∙cm^−3^, meaning it decreased by 0.07 g∙cm^−3^ (Figure 3c), and with the addition of cement, it was on average 1.75 g∙cm^−3^ (Figure 3d) and did not change in relation to the soil without the addition of a binder. The optimum moisture content of coarse silt with added lime was higher by about 1.0 to 1.5% and ranged from 17.0 to 17.7% with the increase in the addition of lime from 3 to 8%, while with the addition of cement, it did not change and was on average 16.2%.

The maximum dry density of the clayey silt with sand was 1.83 g∙cm^−3^ at an optimum moisture content of 13.7% (Figure 3b). The addition of cement caused an average decrease in the maximum dry density of 0.05 g∙cm^−3^, which was on average 1.78 g∙cm^−3^. The optimum moisture content of the clayey silt with sand and 3% to 8% of added cement ranged from 14.7% to 14.1%, so its value increased by 1.0% to 0.4%.

**Figure 4:**
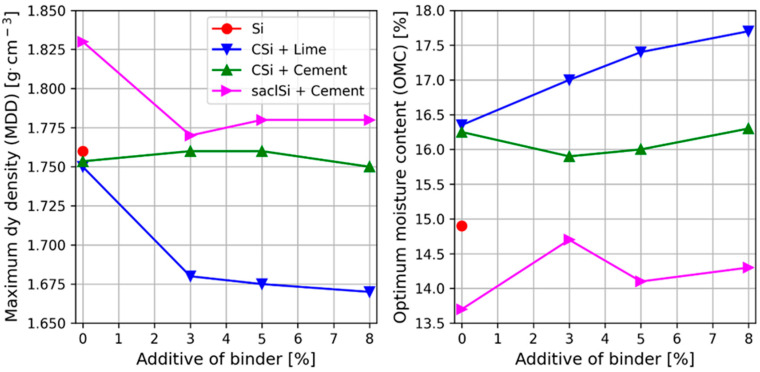
Dependence of the optimum moisture content and maximum bulk density of the skeleton of the tested silty soils on the type and addition of hydraulic binder.

The addition of 3% of lime to coarse silt caused a decrease by about 0.07 g∙cm^−3^ of the maximum dry density and a small increase in the optimum moisture content, i.e., by 1.4% (Figure 4). The effect of a further increase in the addition of lime to 5 and 8% on the value of compaction parameters was not significant. Similar trends were obtained by Bell [14]. Shi et al. [62], in studies of silty clay, showed that with the increase in lime content, the maximum dry density gradually decreased and the optimum moisture content increased. The authors indicate that the optimum compaction conditions were obtained when the addition of lime was 7% [62].

It should be clearly indicated that the addition of cement only in the case of clay–sand silt caused significant changes in the compaction parameters, i.e., a decrease in the maximum dry density of 0.05 g∙cm^−3^ and the optimum moisture content of 1% were obtained. However, in the case of coarse silt, these changes were insignificant. This was caused by differences in the grain size composition of the tested soils. In the case of coarse silt, there was almost two times higher content in the silt fraction and eight times lower content in the sand fraction compared to the clayey silt with sand.

In general, the maximum dry density of the skeleton of the tested soils with the addition of lime was lower in relation to the value for soils with the addition of cement. The maximum dry density of the skeleton of the soil with the addition of binder was lower or the same as that of the soil without its addition. It can also be seen that the optimum moisture content of the tested soils was higher with the addition of lime than with the addition of cement.

### 4.3. Shear Strength

**Figure 5:**
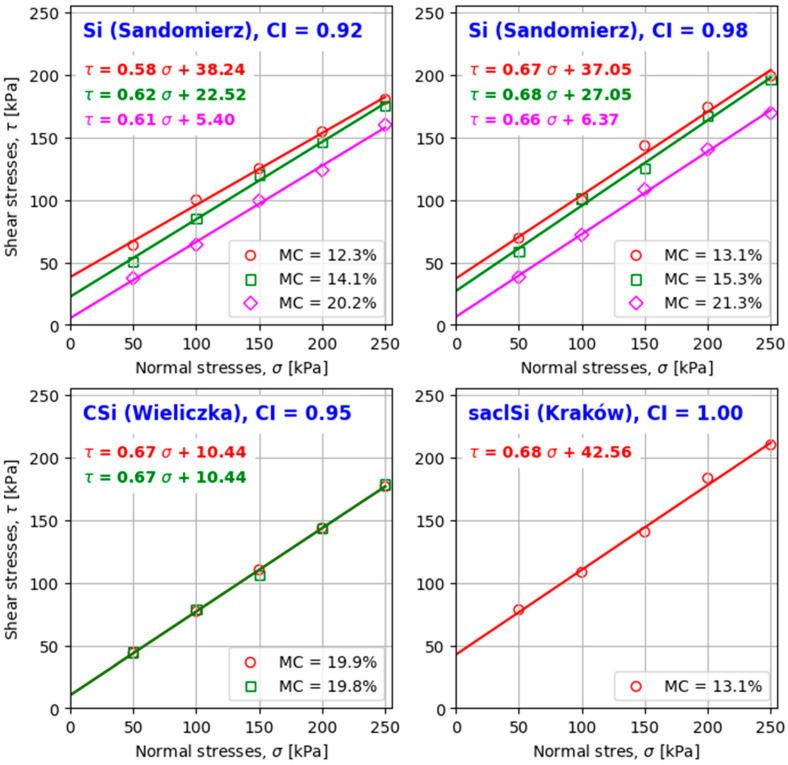
Shear strength line of the tested silty soils without the addition of binder.

**Figure 6:**
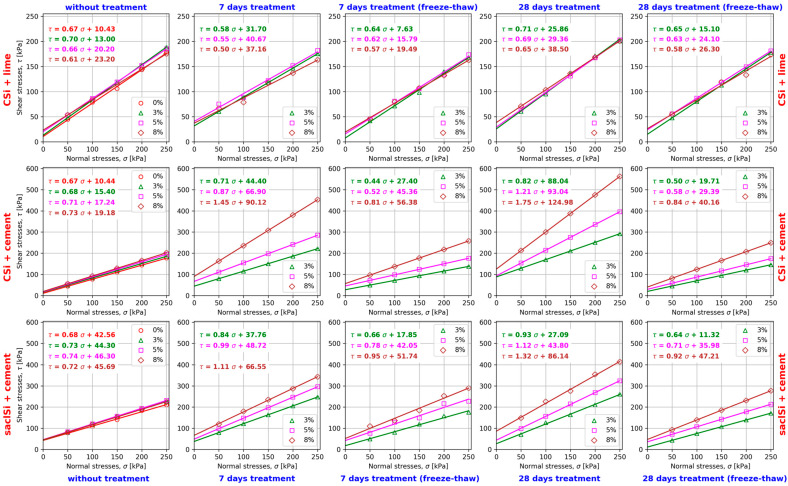
Shear strength line of the tested soils with the addition of binder and without and with treatment.

**Figure 7:**
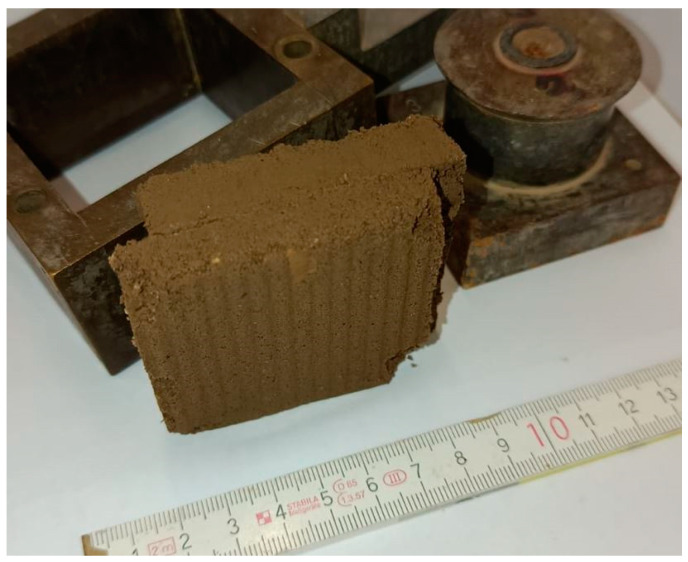
Sample of coarse silt with addition of 3% lime after 7 days of air–water treatment immediately after shearing (visible shear surface) (photo by K. Kamińska).

The obtained values of shear stress depended on the type and addition of hydraulic binder to the tested soil, applied treatment, and normal stresses (Figure 5 and Figure 6). The values of the coefficient of determination R^2^ of the shear strength line were close to or equal to one, which indicates its good fit to the obtained values of shear stress for individual normal stress. Analysis of the dependence of shear stress on horizontal strain allowed us to conclude that plastic shearing was obtained in the majority of the tests. Figure 7 shows a view of the sample immediately after shearing of coarse silt with 5% addition of lime after 7 days of air–water treatment.

In the tests of the Sandomierz silt without the addition of hydraulic binder, the highest shear strength values were obtained for a moisture content lower than optimal, and the lowest values were obtained for a moisture content higher than optimal. Sample compaction was also a significant factor. Higher shear strength values were obtained with a higher compaction index. The decrease in shear strength with moisture content increasing from 12.3 to 20.2% at a compaction index of CI = 0.92 ranged from 11 to 41%, respectively, and at CI = 0.98, it ranged from 15 to 45% at normal stresses of 250 and 50 kPa, respectively. Similar relationships were obtained by, among others, Zydroń and Dąbrowska [63], Zhang et al. [64], and Wang et al. [65].

The comparison of shear strength of silty soils tested at an optimum moisture content showed that the type of soil and thus its grain size composition, as well as compaction, were significant factors influencing the shear strength values. The highest values of shear strength were obtained for clayey silt with sand, for which the tests were performed at a compaction index of CI = 1.00, slightly lower for silt examined at a compaction index of CI = 0.98, and the lowest for coarse silt and silt examined at compaction indexes of CI = 0.95 and 0.92, respectively.

**Figure 8:**
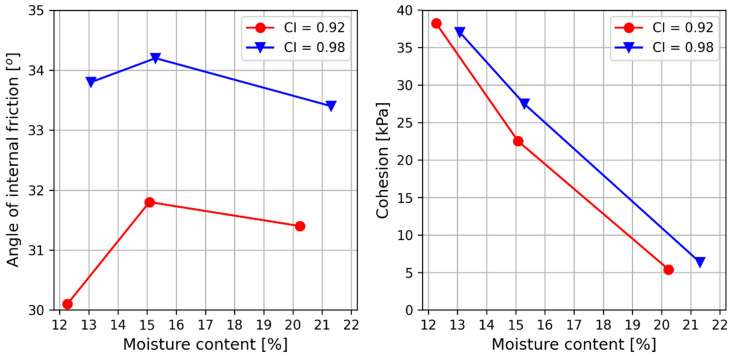
Dependence of the internal friction angle and cohesion on the moisture content of silt.

The tests with the addition of a hydraulic binder showed that the type of binder used, its addition, and the type and time of sample treatment were significant factors affecting the shear strength (Figure 8). The shear strength values were found to be several times higher for soils stabilized with cement than for those stabilized with lime. In this case, the results obtained confirm the relationships obtained from studies of other soils [66,67,68]. The lowest shear strength values were obtained for soil without the addition of binder.

The shear strength analysis of the tested soils without the addition of a binder allowed us to observe that immediately after forming the samples, the shear strength increased slightly, i.e., from 2 to 17 kPa for the adopted normal stresses with an increase in the binder addition from 0 to 8%. A similar relationship was obtained for the tests with the addition of lime. An increase in the addition of lime from 0 to 8% caused fluctuations in the shear strength on average from 42 to 55 kPa and from 163 to 203 kPa for stresses of 50 and 250 kPa, respectively. It should be clearly indicated that in the case of normal stresses of 50 kPa, an increase in the shear strength from about 4 to 10 kPa was obtained, and at normal stresses of 250 kPa, it decreased by 12 kPa with an increase in the addition of lime.

In the case of tests of coarse silt and clay–sand silt with the addition of cement from 3 to 8%, significant differences in shear strength values were obtained. The greatest increase in shear strength was obtained in tests of coarse silt after 7 and 28 days of air–water treatment. In this case, the addition of cement caused an increase in shear strength of about 84 kPa and 231 and 271 kPa, respectively, at normal stresses of 50 and 250 kPa. In the case of tests using 3 and 14 freeze–thaw cycles, the increase in shear strength by 47 and 37 kPa with the addition of cement was obtained, respectively, for normal stresses of 25 kPa and by 120 and 104 kPa for normal stresses of 250 kPa.

In the tests of clay–sand silt, after 7 and 28 days of air–water treatment, the increase in the shear strength value was much smaller and amounted to 40 and 78 kPa and 95 and 153 kPa, respectively, at normal stresses of 25 and 250 kPa. In the tests, in which 3 and 14 freeze–thaw cycles were applied, the increase in the shear strength was 60 and 50 kPa and 114 and 106 kPa, respectively, at normal stresses of 25 and 250 kPa, i.e., with clearly weaker effects of the cement addition on the shear strength values.

### 4.4. Shear Strength Parameters

The values of the shear strength parameters of the Sandomierz silt were significantly dependent on the compaction and moisture content at which the samples were sheared (Table 4). The increase in compaction from CI = 0.92 to 0.98 caused the angle of internal friction to increase from 2° to 4° and the cohesion to decrease from 1 to 5 kPa (Figure 8). On the other hand, the increase in moisture content from 12% (smaller than optimal) to 15% (close to optimal) caused the internal friction angle to increase from about 0.5 to almost 2°, respectively, at compaction indexes of CI = 0.92 and 0.98. A further increase in moisture from about 21% (above optimal) caused the internal friction angle to decrease by an average of 0.6° for both compaction values. In the case of cohesion, the moisture content increase caused the cohesion to decrease by 33 and 31 kPa with compaction indexes of CI = 0.92 and 0.98, respectively.

Similar relationships related to the effects of moisture on shear strength parameters were obtained by, among others, Bláhová et al. [69], Zheng et al. [70], Kang et al. [67], and Wang et al. [65]. In their studies, these authors showed that the soils they tested tended to strengthen with decreasing moisture contents, which means their angle of internal friction and cohesion increased. In addition, the water film between soil particles gradually changed from continuous to intermittent, which caused its shrinkage [65]. The authors clearly indicated the significant importance of the moisture content on the cohesion values. In the case of the angle of internal friction, changes in its value were recorded, but to a much smaller extent.

The conducted studies indicated the need to limit the influence of moisture content on shear strength parameters. These activities can be carried out using various methods, including draining of the area, freezing the soil or surface, or deep stabilization using hydraulic binders.

**Figure 9:**
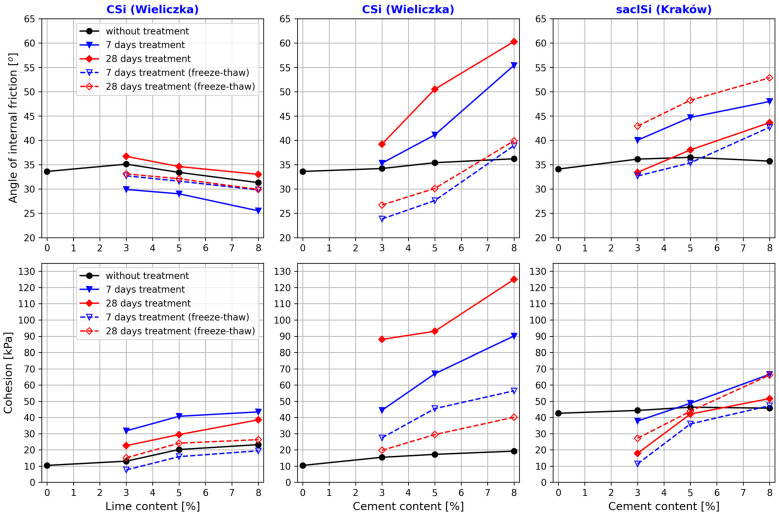
The effects of the type and addition of binder and the type and time of treatment on the values of internal friction angle and cohesion of the tested silty soils.

When analyzing the values of the shear strength parameters of the tested soils with the addition of lime and cement, it should be clearly indicated that they differ significantly depending on the type and addition of the binder (Table 5 and Table 6, Figure 9). Other factors influencing the values of the angle of internal friction and soil cohesion were the type and time of sample treatment.

The shear strength parameters of the silty soils tested immediately after formation showed minor changes. In the case of adding lime to the coarse silt, a decrease in the value of internal friction by about 2° and an increase in cohesion by about 13 kPa was observed. The addition of cement to the coarse silt caused an increase in the angle of internal friction by about 2° and cohesion by 9 kPa. In the case of clay–sandy silt, no significant changes in the angle of internal friction and cohesion were observed. The changes observed were caused by the formation of chemical bonds during shearing of the stabilized soil samples.

When analyzing the effects of lime addition to coarse silt, it should be noted that increasing the lime content from 3% to 8% caused a decrease in the internal friction value on average by about 4° (from 10% to 15% relative) for the air–water treatment and by about 3° (10% relative) for the treatment of samples with freeze–thaw cycles (Table 4, Figure 9). In the case of cohesion, an inverse relationship was obtained. Increasing the lime addition caused an increase in cohesion by 12 kPa and 16 kPa (37% and 70% relative) for 7 and 28 days of air–water treatment, respectively. The use of treatment with freeze–thaw cycles together with an increase in the lime addition caused an increase in cohesion on average by about 12 kPa, which was from 155% to 75% relative for 3 and 14 freeze–thaw cycles, respectively.

Taking into account the type of treatment, it was found that the highest values of the angle of internal friction for coarse silt with lime addition were obtained after 28 days, and the lowest were obtained after 7 days of air–water treatment. The values of the angle of internal friction for the samples with the freeze–thaw cycles were very similar, but they were lower by about 2° compared to the samples sheared immediately after forming. The angle of internal friction of the tested soil after 7 days of air–water treatment decreased on average by 5° (16% relative), and after 28 days, it increased on average by 1.5° (5% relative) compared to the tests conducted immediately after forming the samples. In the case of treatment with freeze–thaw cycles, a decrease in the angle of internal friction was obtained by almost 2° (6% relative) compared to the tests conducted immediately after forming the samples. The increase in lime addition caused the internal friction angle to decrease by an average of 4° (12% relative) and by 3° (10% relative) in the tests after treatment without and with freeze–thaw cycles, respectively.

The highest cohesion values of coarse silt with lime addition were obtained after 7 days of air–water treatment, and the lowest cohesion values were also obtained after 7 days of treatment but with three freeze–thaw cycles. The values differed on average by 24 kPa. The cohesion of the tested soil after 7 and 28 days of air–water treatment increased on average by 20 kPa (111% relative) and 11 kPa (62% relative) in relation to the tests conducted directly after the samples were formed. In the case of treatment with 3 and 14 freeze–thaw cycles, a decrease in cohesion of almost 5 kPa (27% relative) was obtained, and it increase by about 3 kPa (16% relative) in relation to the tests conducted directly after the samples were formed. The increase in lime addition resulted in an increase in cohesion by an average of 13 kPa (37%—3 cycles to 70% relative—14 cycles) and 11 kPa (74%—3 cycles to 155% relative—14 cycles) in the tests after treatment without and with the freeze–thaw cycles, respectively.

Different results of the tests of the shear strength parameters of silty clay with lime addition were obtained by Shi et al. [62]. In their study, the addition of lime improved the internal friction and cohesion values. Cohesion increased with the addition of lime, while the relationship between the angle of internal friction and the addition of lime was insignificant. The treatment conditions had a significant effect on the mechanical properties of the modified soil. With a small addition of lime (3%), water treatment worsened the shear strength of the stabilized soil, while with a large addition of lime (7% and 9%), the stabilized soil was more resistant to water treatment. It was also shown that the modified silty clay showed greater shear strength at higher normal stress values [62].

The highest internal friction and cohesion values of the coarse silt with added cement were obtained for tests conducted after 28 days of the air–water treatment. Lower values of the tested parameters were obtained after 7 days of the air–water treatment. The lowest values of internal friction angle were obtained for the samples treated with 3 freeze–thaw cycles, and the lowest cohesion values were obtained after 14 such cycles. In the case of cohesion, all its values, regardless of the type and time of treatment, were higher than the cohesion in tests performed directly after the samples were formed. The addition of cement from 3% to 8% to coarse silt from Wieliczka in the case of 7 days and 28 days of air–water treatment caused an increase in the value of internal friction angle from 1° to 19° (from 3% to 53% relative) and from 5° to 24° (15% to 67% relative) compared to the tests performed immediately after the samples were formed. However, when applying the treatments with 3 and 14 freeze–thaw cycles, the obtained internal friction angle values were lower by 10° and 8° (30% and 22% relative) with 3% cement addition, by about 8° and 5° (22% and 15% relative), and higher by about 3° and 4° (7% and 10% relative) with 8% cement addition compared to the tests performed immediately after the samples were formed. An increase in the added cement from 3% to 8% in the tested coarse silt caused an increase in the internal friction angle by an average of 21° (55% relative) in the case of the air–water treatment and by an average of 14° (from 49%—14 cycles to 63% relative—3 cycles) after treatment with the freeze–thaw cycles. The addition of cement from 3% to 8% increased the cohesion from 29 kPa to 71 kPa (from 188% to 369% relative) and from 73 kPa to 106 kPa (from 471% to 551% relative) after 7 and 28 days of the air–water treatment, respectively, compared to the tests conducted immediately after the samples were formed. The treatment with freeze–thaw cycles increased the cohesion from 12 kPa to 37 kPa (from 78% to 194% relative) and from 4 kPa to 21 kPa (from 28% to 109% relative) with 3 and 14 freeze–thaw cycles, respectively, in comparison to the tests conducted immediately after the samples were formed. When analyzing the effects of cement addition on the cohesion values, it should be clearly noted that the increase in its value was 46 kPa and 37 kPa (103% and 42% relative) after 7 days and 28 days of treatment, respectively, and 29 kPa and 20 kPa (105% relative on average) after treatment with 3 and 14 freeze–thaw cycles, respectively.

When comparing the effects of lime and cement addition on the values of internal friction angle and cohesion of coarse silt from Wieliczka, it can be stated that the addition of cement allowed us to obtain significantly higher values of these parameters regardless of the amount of binder added, as well as the type and time of treatment. An increase in the amount of binder added from 3 to 8% in the case of cement caused an increase in the values of both parameters tested, and in the case of lime, a decrease in the angle of internal friction and an increase in cohesion.

Slightly different results were obtained in the study of clayey silt with sand from Krakow with the addition of cement. Similarly to the case of coarse silt from Wieliczka, the angle of internal friction and cohesion increased with the increase in the cement addition, but the increase in their values was much smaller.

The addition of cement from 3% to 8% to the clayey silt with sand in the case of the 7-day and 28-day air–water treatment caused an increase in the value of the internal friction angle from 4° to 12° (from 11 to 34% relative) and from 7° to 17° (19% to 48% relative) compared to the tests conducted immediately after the samples were formed. On the other hand, when treatment with 3 and 14 freeze–thaw cycles was used, the internal friction angle values were lower by about 3° (9% relative) with 3% cement addition, similar values were obtained for 5% cement addition, and higher values—by about 7.5° (20% relative)—were obtained for 8% cement addition compared to the tests conducted immediately after the samples were formed. The increase in the cement added to the tested soil caused the internal friction angle to increase on average by 9° (22% relative) and by 10° (31% relative) in the tests without and with freeze–thaw cycles, respectively.

However, the highest cohesion values were obtained after 7 days of the air–water treatment, and the lowest were obtained after 28 days of treatment with 14 freeze–thaw cycles. It is worth noting that the cohesion values obtained in the tests conducted after 7 and 28 days of air–water treatment and after 3 and 14 freeze–thaw cycles were similar.

Compared to the tests performed immediately after sample formation, the cohesion of clay–sand silt after 7 days and 28 days of air–water treatment decreased from 7 kPa to 17 kPa (15% and 39% relative) with 3% cement addition. This was similar with 5% cement addition and higher by 21 kPa (from 5% relative) with 8% cement addition. By using 3 and 14 freeze–thaw cycles, the cohesion value decreased by about 26 kPa and 33 kPa (60% and 75% relative) afor3% cement addition; with 5% addition, the decrease was 4 kPa and 10 kPa (9% and 22% relative) and increased by 6 kPa and 2 kPa (13% and 3% relative) with 8% cement addition. When analyzing the effects of cement addition from 3% to 8% on the cohesion values, its value increased by 29 kPa and 39 kPa (76% and 144% relative) after 7 days and 28 days of treatment, respectively, and 34 kPa and 36 kPa (189% and 317% relative) after treatment with 3 and 14 freeze–thaw cycles, respectively.

In summary, it should be noted that the changes in strength parameters with the increase in the binder addition to the tested soil maintained the same trend of decreasing or increasing their values, regardless of the type or time of treatment.

The comparison of the effects of cement addition on the strength parameter values of coarse silt and clayey silt with sand shows that slightly higher values of the internal friction angle and almost twice as high cohesion values were obtained for the coarse silt. It should also be emphasized that no effects of compaction on the tested parameters were found. The coarse silt tests were conducted with a lower compaction than in the case of clayey silt with sand tests.

Similar relationships were obtained for the addition of cement to marine sediments by Furlan et al. [71]. The use of binder, along with the extension of setting time, had a positive effect on cohesion. The increase in cohesion for the samples stabilized with cement or fly ash of marine sediments was the result of chemical bonding and capillary bridging strengthened additionally by the setting time. The cohesion of all the stabilized samples was generally higher than that of the unstabilized samples. The results also show that the angle of internal friction did not change, and its value was close to the value for the untreated sediment. Similarly, Ashiq et al. [25], in their analyses using the finite element method, demonstrated the effectiveness of cement stabilization of soil in mitigating long-term subsidence and increasing the stability of embankments in Teknaf regions in Bangladesh. The conducted studies showed that, compared to natural soil conditions, cement-stabilized soil significantly reduces long-term settlement of the embankment base. These authors also showed that 4% cement addition was optimal to maintain a balance between cost-effectiveness and performance.

Interesting results of research on the shear strength parameters of cement-reinforced sands were obtained by Boutouba et al. [72]. The research results showed that the extension of the treatment time of the samples caused an increase in the shear strength and, consequently, also an increase in the angle of internal friction and cohesion, obtaining their maximum values at 10% cement addition. The authors showed that the increase in moisture content significantly increased the shear strength of the sand–cement mixture due to the hydration of cement.

As demonstrated, the addition of lime and cement to mineral soils, including silty soils, is widely used in civil engineering for surface and subsurface stabilization. However, the use of this type of reinforcement with hydraulic binders depends on a number of factors related to the geotechnical characteristics of the reinforced soil, and also on climatic conditions. Cyclic freezing and thawing of the soil causes significant weakening of the soil with the addition of binder, which was observed in the conducted studies and is also described in the literature [73,74].

It should also be pointed out that many times during the construction or modernization of a communication route, when cohesive or heave soils were encountered, the only solution was to replace the soil with one that was easy to compact and had the desired load-bearing capacity. The method is effective but expensive and time-consuming. The extraction and movement of huge masses of earth is always associated with far-reaching, negative interference with the natural environment. This is related to the need to use trucks, which can contribute to the rapid degradation of local roads, becoming a great nuisance to residents. It should also be remembered that the cohesive, non-load-bearing soil extracted as a result of the exchange is treated as unnecessary and burdensome waste, for which a place must be found for storage.

### 4.5. Numerical Modeling Analysis

**Figure 10:**
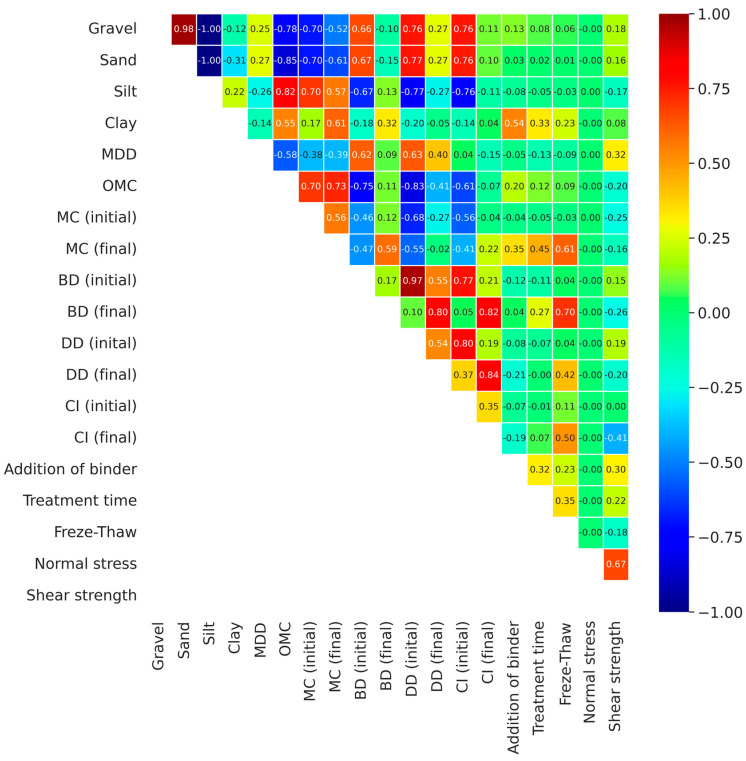
Correlation matrix of measured parameters. Gravel, Sand, Silt, Clay [%]—fraction content, MDD [g∙cm^−3^]—maximum dry density, OMC [%]—optimum moisture content, MC [%]—moisture content, D [g∙cm^−3^]—bulk density, DD [g∙cm^−3^]—dry density, CI [-]—compaction index, Addition of binder [%], Treatment time [days], Freeze–Thaw Cycles [times], Normal stress [kPa], Shear stress (strength) [kPa], initial, final—before, after tests.

For the obtained research results, a correlation matrix of the tests of each independent variable is presented in Figure 10. Based on the analysis of the correlation matrix in terms of the shear strength, it is clear that there is a strong positive correlation with normal stress (0.67), as well as with the maximum bulk density (0.32) and the amount of binder addition (0.30) (Figure 10). On the other hand, a strong negative correlation occurred with respect to the compaction index after the test (−0.41) and the bulk density after the test (−0.26) and initial moisture content (−0.25).

In the next stage of the analysis, shear strength calculations were performed based on the input parameters using the XGBoost method, which is classified as a supervised machine learning method. In order to fit the model and prevent its overfitting, part of the data were omitted at the model training stage, and a blind sample was used to test the obtained results.

**Figure 11:**
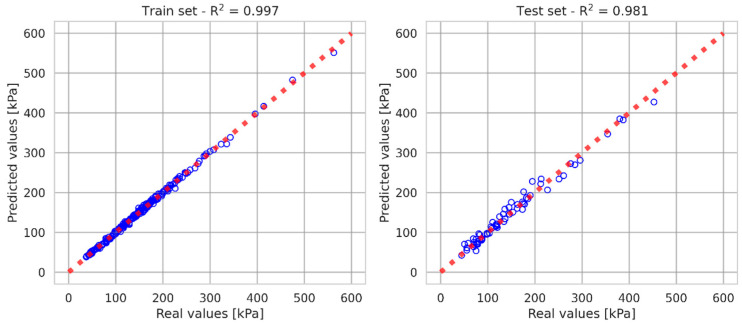
Comparison of shear strength values obtained from tests and those predicted using XGBoost.

The influence of individual factors on the shear strength test results were determined using the XGBoost gradient boosting algorithm, then the SHAP value for each independent variable was determined. Figure 11 presents the results of shear strength prediction of soil related to the values obtained from direct tests. The obtained values of the coefficient of determination R^2^ indicate that the applied calculation model enables correct prediction of shear strength for the obtained results using the output data.

The prediction model was built with many input parameters, and the output parameter was shear strength. The modeling results showed that the XGBoost model had a high ability to accurately predict the shear strength. In addition, XGBoost has the advantage of being easy to update, which makes the proposed model open to further development. Collecting more data will lead to a much stronger prediction ability, avoiding the requirement of expertise and time to update the existing design aid. This is also confirmed by the studies of other researchers (e.g., [53,75]).

**Figure 12:**
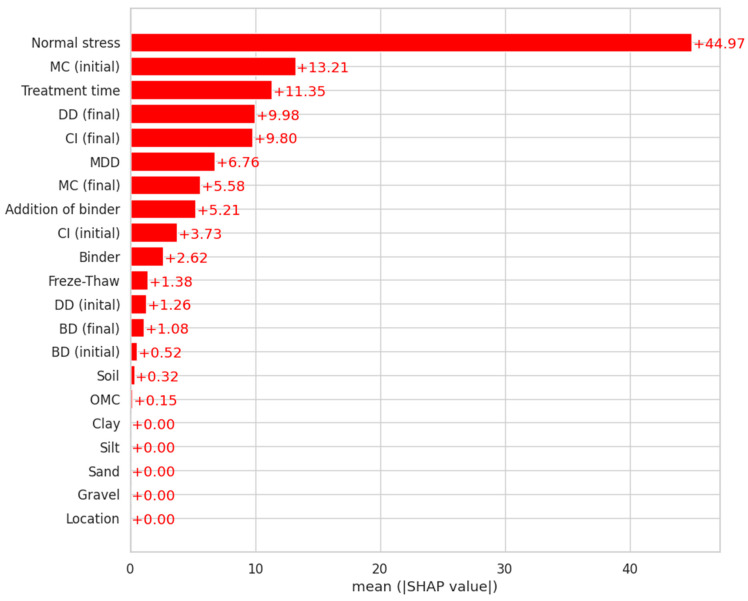
Graphical analysis of results for averages of SHAP values (legend description of the vertical axis the same as for Figure 10).

The graph of the shear strength prediction results, which is a global interpretation of the model, is shown in Figure 12, where the mean SHAP values, i.e., the influence of the average feature on the model result, are presented. The calculations of the Shapley values showed that normal stresses, characterized by the most significant degree of importance of the feature parameters, were the main parameter determining the predicted shear strength values. The important explanatory parameters also included the initial soil moisture content and sample treatment time (SHAP > 10). The bulk density and the compaction index were also characterized by the high feature importance (SHAP > 9). On the other hand, the location of the soil sampling site and the content of gravel, sand, silt, and clay fractions had the value of SHAP = 0, which indicates that they were not important for predicting the shear strength value.

**Figure 13:**
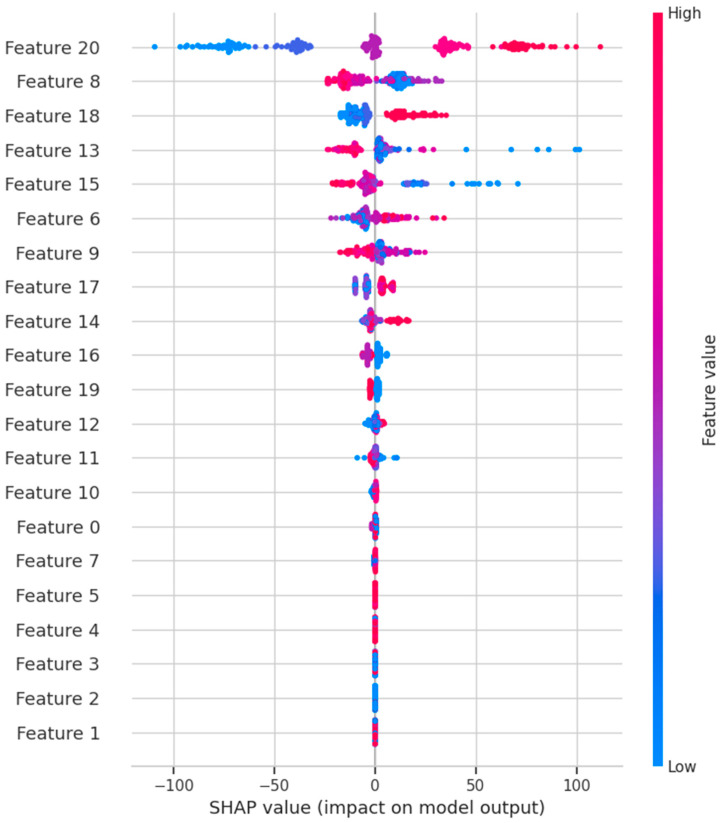
SHAP value bee graph: Feature 0—kind of soil; Feature 1—location of soil collection; Feature 2—content of gravel fraction [%]; Feature 3—content of sand fraction [%]; Feature 4—content of silt fraction [%]; Feature 5—content of clay fraction [%]; Feature 6—MDD, maximum dry density [g∙cm^−3^]; Feature 7—OMC, optimum moisture content [%]; Feature 8—MC, initial moisture content [%]; Feature 9—MC, final moisture content [%]; Feature 10—BD, initial bulk density before [g∙cm^−3^]; Feature 11—BD, final bulk density [g∙cm^−3^]; Feature 12—DD, initial dry density [g∙cm^−3^]; Feature 13—DD, final density after [g∙cm^−3^]; Feature 14—CI, initial compaction index [-]; Feature 15—CI, final compaction index [-]; Feature 16—type of binder; Feature 17—Addition of binder [%]; Feature 18—care time [days]; Feature 19—freeze–thaw cycles [times]; Feature 20—normal stress [kPa].

In Figure 13, the independent variables are arranged in descending order based on the relative importance of each feature $\sum_{j=1}^{N}\left|\emptyset_{i}^{\left(j\right)}\right|$, where $\emptyset_{i}$ is the Shapley value of parameter *i*, *j* is the sample, and *N* is the total number of samples. Each point in the figure represents a sample relative to its influence on the model output $\emptyset \left(j\right)$. The color of each sample represents the relative value of parameters *i* from low to high [76,77]. According to the distribution, the most important parameter was normal stress. The horizontal spread of the SHAP values indicates the change in the parameter values. The larger the spread, the greater the change in the parameter and therefore the greater the importance of the covariate. Covariates were ranked from most to least influential according to their mean absolute SHAP value.

**Figure 14:**
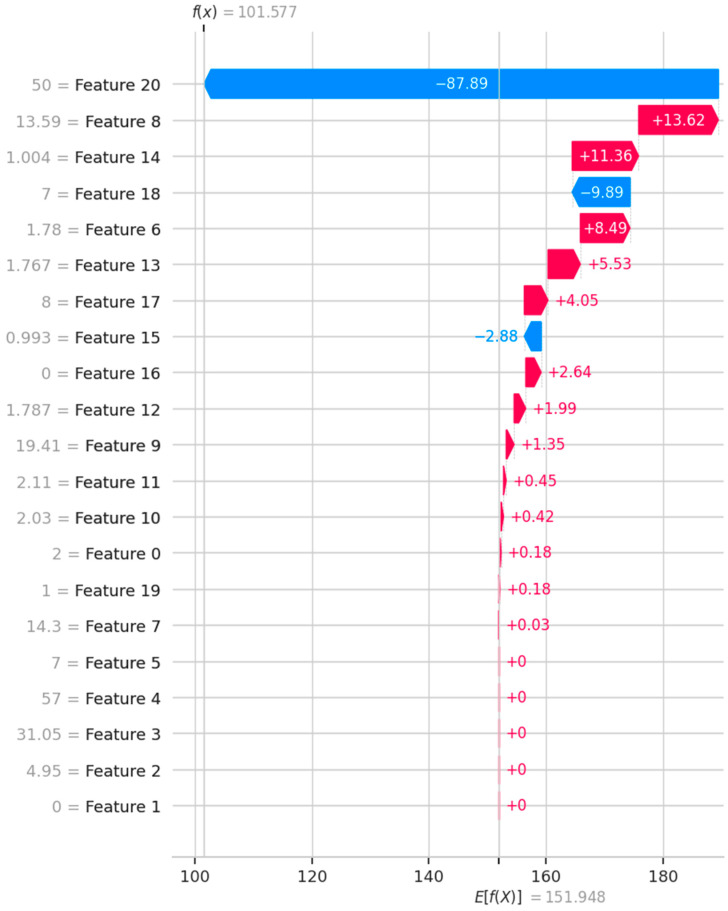
Waterfall graph of SHAP values (legend description of the vertical axis the same as for Figure 13).

Figure 14 shows the SHAP value for each independent variable. The vertical dashed line indicates the expected SHAP value. The SHAP values add up to the final model prediction. By analyzing Figure 14, the exact change in shear strength resulting from the inclusion of each variable can be determined.

Most of the existing machine learning models for assessing the geotechnical properties of soils are of a black box nature [78,79]. In the conducted analyses, an attempt was made to consider a number of factors affecting the shear strength by taking into account the SHAP method. Based on the interpretation of the results obtained using the SHAP algorithm, it was observed that normal stress, initial moisture content, and curing time of the samples were the main input features that affected the shear strength, indicating that their higher values increase the model output.

## 5. Conclusions

The conducted tests of the compaction and shear strength parameters of the selected silty soils stabilized with the addition of lime and/or cement showed significant differences in the examined parameters. Shear strength tests conducted using a direct shear apparatus showed an increase in the shear strength parameters of soils with the addition of a binder depending on the amount added. The research carried out showed that the use of hydraulic binders reduces the need to replace moist and non-bearing soils and thus reduces the impact on the natural environment.

The compaction curves of the tested silty soils with the addition of lime and/or cement obtained using the Proctor apparatus using the standard method were characterized by a shape similar to the curves for the soils without the addition of binder. The addition of lime caused a significant decrease in the maximum bulk density of the skeleton and a slight increase in the optimum moisture content of coarse silt compared to the soil without the addition of lime. The effects of cement were, however, ambiguous. In the case of coarse silt, the changes were small, while for clay–sand silt a decrease in both tested parameters was observed. The effects of the amount of lime or cement addition on the values of the maximum bulk density of the skeleton and optimum moisture content were small. It was found that the maximum bulk density of the tested soils with the addition of lime was lower, and the optimum moisture content was higher for the adopted addition of lime than for the same addition of cement.

The shear strength values of the tested silty soils depended on the type of binder as well as on the type and time of consolidation. The effect of the binder type depended on the type and time of treatment. In general, the tests of the soils with the binder addition after the air–water treatment provided higher values of shear strength parameters, while after the freeze–thaw cycles, these values were smaller. A reduction in shear strength was observed after the freeze–thaw cycles, along with a decrease in the angle of internal friction and cohesion. Increased addition of lime caused a decrease in the angle of internal friction and increase in cohesion. On the other hand, the addition of cement in the case of both tested soils caused an increase in the value of the angle of internal friction and cohesion.

The shear strength was positively correlated with normal stress and negatively correlated with initial moisture content and compaction. The sensitivity analysis confirmed that the greatest influence on the obtained shear strength values was exerted by the value of the normal stress. The obtained shear strength values were further affected by the initial moisture content as well as the type and time of treatment. As reflected in the SHAP values, the content of individual fractions in the tested soils was of negligible importance in the case of the conducted tests.

Further research will be carried out to increase the size of the database and also to extend the range of input features by including other hydraulic binders dedicated to soil stabilization produced, e.g., on the basis of fly ash. In models using machine learning, data are crucial to construct a reliable predictive tool. A wider range of input data may increase the predictive power of future models predicting the shear strength of soils stabilized with hydraulic binders.

## Figures and Tables

**Table 1:** Characteristics of treatment conditions of silty soil samples with the addition of a hydraulic binder.

Treatment Time [Days]	Addition of Lime or Cement [%]	Type of Sample Treatment	Number of Samples for Each Binder Addition	Sample Treatment Description
0	0	Without treatment	4	Shear strength determination immediately after forming the samples.
	3			
	5			
	8			
7	3	Air–water treatment	4	3 days at room temperature protected from drying out, 1 day immersed in water to a height of 1 cm, 3 days completely immersed in water.
	5			
	8			
28	3		4	13 days at room temperature protected from drying out, 1 day immersed in water to a height of 1 cm, 14 days completely immersed in water.
	5			
	8			
7	3	Air–water treatment with freeze–thaw cycles	4	3 days at room temperature protected from drying out, 1 day completely immersed in water, 3 days with freeze–thaw cycles.
	5			
	8			
28	3		4	13 days at room temperature protected from drying out, 1 day completely immersed in water, 14 days with freeze–thaw cycles.
	5			
	8			

**Table 2:** Geotechnical properties of the tested soils.

Parameter			Unit	Location of Soil Collection		
				Sandomierz	Wieliczka	Kraków
				Value		
Fraction content	Gravel, Gr: 63 ÷ 2 mm		%	0	0	4.95
	Sand, Sa: 2 ÷ 0.063 mm			8.39–10.5	1.26–1.30	31.05
	Silt, Si: 0.063 ÷ 0.002 mm			85.1–87.11	89.70–92.24	57.00
	Clay, Cl: <0.002 mm			4.85–4.50	6.50–9.00	7.00
Name of soil according to [44]				Silt (Si)	Coarse silt (CSi)	Clayey silt with sand (saclSi)
Equivalent diameter		d_60_	mm	0.30	0.032	0.045
		d_30_		0.0175	0.0017	0.0175
		d_10_		0.0042	0.0026	0.0035
Uniformity coefficient, $C_{U}=\frac{d_{60}}{d_{10}}$			-	7.0	15.0	14.3
Coefficient of curvature, $C_{C}=\frac{d_{30}^{2}}{d_{10}\cdot d_{60}}$			-	2.43	4.82	1.85
Density of solid particles, *ρ*_s_			g∙cm^−3^	2.67	2.67	2.69

**Table 3:** Geotechnical properties of the tested soils.

Name of Soil	Location of Soil Collection	Binder		Maximum Dry Density (MDD) [g∙cm^−3^]	Optimum Moisture Content (OMC) [%]
		Type	Additive [%]		
Silt (Si)	Sandomierz	Without	0	1.76	14.9
Coarse silt (CSi)	Wieliczka	Without	0	1.75 and 1.76 mean 1.75	15.9 and 16.6 mean 16.3
		Lime	3	1.68	17.0
			5	1.68	17.4
			8	1.67	17.7
		Cement	3	1.75	16.2
			5	1.76	16.0
			8	1.75	16.3
Clayey silt with sand (saclSi)	Kraków	Without	0	1.83	13.7
		Cement	3	1.77	14.7
			5	1.78	14.1
			8	1.78	14.3

**Table 4:** Comparison of the results of the internal friction angle and cohesion of silt (Si) from Sandomierz—test without binder addition.

**Parameter**	**Unit**	**Value**					
Compaction index (CI)	-	0.92			0.98		
Moisture content—description	-	$w<w_{opt}$	$w=w_{opt}$	$w>w_{opt}$	$w<w_{opt}$	$w=w_{opt}$	$w>w_{opt}$
Moisture content after test	%	12.3	15.1	20.2	13.1	15.3	21.3
Angle of internal friction	°	30.1	31.8	31.4	33.8	34.2	33.4
Cohesion	kPa	38.2	27.5	6.4	37.1	22.5	5.4

**Table 5:** Comparison of the results of the internal friction angle and cohesion of coarse silt (CSi) from Wieliczka from tests with and without the addition of a binder.

Binder Additive	Treatment Time	Moisture Content After Test	Angle of Internal Friction	S Cohesion	Moisture Content After Test	Angle of Internal Friction	Cohesion	Moisture Content After Test	Angle of Internal Friction	Cohesion	Moisture Content After Test	Angle of Internal Friction	Cohesion
%	days	%	°	kPa	%	°	kPa	%	°	kPa	%	°	kPa
Binder		Lime						Cement					
Type of treatment		Immediately after forming the sample						Immediately after forming the sample					
0	0	16.8	33.6	10.4				16.9	33.6	10.4			
3		17.3	35.1	13.0				16.5	34.2	15.4			
5		17.6	33.4	20.2				16.2	35.4	17.2			
8		18.2	31.3	23.2				16.1	36.2	19.2			
Type of treatment		Air–water treatment			Air–water treatment with 3 freeze–thaw cycles			Air–water treatment			Air–water treatment with 3 freeze–thaw cycles		
3	7	16.7	29.9	31.7	16.9	32.7	7.6	16.1	35.3	44.4	16.1	23.8	27.4
5		17.7	29.0	40.7	17.2	31.6	15.8	15.9	41.1	66.9	15.3	27.6	45.4
8		17.8	25.5	43.4	17.7	29.8	19.4	15.8	55.4	90.1	15.7	38.9	56.4
Type of treatment		Air–water treatment			Air–water treatment with 14 freeze–thaw cycles			Air–water treatment			Air–water treatment with 14 freeze–thaw cycles		
3	28	16.5	36.7	22.6	16.9	33.1	15.1	15.8	39.2	88.0	16.9	26.7	19.7
5		16.8	34.6	29.4	17.3	24.1	24.1	15.7	50.5	93.0	16.6	30.1	29.4
8		17.3	33.0	38.5	17.7	26.3	26.3	16.6	60.3	125.0	15.6	39.9	40.1

**Table 6:** Comparison of the results of the angle of internal friction and cohesion of clayey silt with sand (saclSi) from Kraków from tests with and without the addition of a binder.

Binder Additive	Treatment Time	Moisture Content After Test	Angle of Internal Friction	Cohesion	Moisture Content After Test	Angle of Internal Friction	Cohesion
%	days	%	°	kPa	%	°	kPa
Binder		Cement					
Type of treatment		Immediately after forming the sample					
0	0	13.7	34.1	42.6			
3		14.3	36.1	44.3			
5		14.1	36.5	46.3			
8		13.9	35.7	45.7			
Type of treatment		Air–water treatment			Air–water treatment with 3 freeze–thaw cycles		
3	7	14.1	40.0	37.8	14.0	33.4	17.9
5		13.6	44.7	48.7	13.6	38.0	42.1
8		13.6	48.0	66.6	13.6	43.6	51.6
Type of treatment		Air–water treatment			Air–water treatment with 14 freeze–thaw cycles		
3	28	14.2	42.9	27.1	14.3	32.7	11.3
5		13.6	48.2	43.8	13.8	35.4	36.0
8		13.7	52.9	86.1	14.1	42.7	47.2

## Data Availability

The original contributions presented in this study are included in the article. Further inquiries can be directed to the corresponding author(s).
